# Estrogen-regulated PTTG1 promotes breast cancer progression by regulating cyclin kinase expression

**DOI:** 10.1186/s10020-020-00161-7

**Published:** 2020-04-09

**Authors:** Chunhui Meng, Yan Zou, Weiwei Hong, Chunhua Bao, Xiaofeng Jia

**Affiliations:** 1 Department of General Surgery, Heze Municipal Hospital, Caozhou Road, Heze, 274000 Shandong China; 2 Department of Oncology, Heze Municipal Hospital, Caozhou Road, Heze, 274000 Shandong China

**Keywords:** Estrogen, Breast cancer, PTTG1, *CCNA2*, *CCNB2*

## Abstract

**Background:**

The present study aims to investigate the effects of pituitary tumor transforming gene (PTTG) 1 on breast cancer and its underlying mechanism.

**Methods:**

GEO data set was applied to analyze the relationship between PTTG1 and survival status and the TCGA breast cancer dataset was used to explore its possible targets. The stable cell lines including PTTG1 knockdown cells, estrogen receptor (ESR) 1 knockdown cells, and PTTG1 overexpression cells were constructed. MTT was used to determine cell viabilities. Propidium iodide (PI) staining and flow cytometry were used to analyze the cell cycle. Quantitative polymerase chain reaction (qPCR) was employed to determine the mRNA expressions. Points mutations and luciferase reporter assays were used to determine the binding sites of estrogen.

**Results:**

PTTG1 was associated with poor survival rates in breast cancer. In vitro study demonstrated that PTTG1 affected cell viabilities of MCF7 and T47D cells. Besides, PTTG1 affected cell cycle arrest of breast cancer cells. Overexpression of PTTG1 led to more breast cancer cells distributed in S phase. The levels of PTTG1 were associated with estrogen and further results showed that the levels of PTTG1 were positively correlated to tamoxifen resistance. Two genes including *CCNA2* and *CCNB2* were identified to be possible targets of *PTTG1*.

**Conclusion:**

Estrogen-regulated PTTG1 promotes the development of breast cancer cells by the regulation of the cell cycle.

## Introduction

Breast cancer is one of the most commonly occurring cancers in women and the death rates of breast cancer in women are ranked as 2nd worldwide (Siegel et al. 2019). Breast cancer also occurs in men although it is very rare (Gucalp et al. 2019). In 2019, there are estimated 268,600 women will be diagnosed as breast cancer in the United States, which accounts for 30% of newly diagnosed cancers (Eismann et al. 2019; Lin et al. 2019). Additionally, there will be 42,260 deaths caused by breast cancer in 2019 (Lin et al. 2019). Studies have identified many risk factors for breast cancers including age, family history, inherited mutations, and hormone replacement therapy (Friedenreich 2001; Hulka and Moorman 2008). When the tumor only locates in the breast, the 5-year survival rate is 99%, whereas 5-year survival rates are declined to 85% if metastasis occurs in regional lymph nodes and to 27% once the tumor spreads into other parts of the body (Carter et al. 1989; Giordano et al. 2004). The low survival rate of patients with breast tumor metastasis is in part due to late diagnosis, which is also a major challenging in the treatment of breast cancer. Therefore, it is important to identify prognostic markers of breast cancer.

Estrogens are natural hormones known to play important roles in the development of sexual and other body functions (Gruber et al. 2002). There is a connection between estrogen and breast cancer, supported by many studies that have reported the levels of estrogen associated with the occurrence and development of breast cancer (Blander 2006; Campagnoli et al. 1999; Guo et al. 2018). It is reported that about 80% of patients with breast cancer are also estrogen receptor (ER)-positive (Blander 2006). Additionally, long-time exposure to estrogen is one of the risk factors for breast cancer. The mechanisms underlying estrogens in breast cancer are in part mediated by the estrogen receptor signaling pathways (Blander 2006). Estrogen receptor signaling pathways induce the production of oxidative metabolites and the mutations of genes, therefore contributing to the DNA damage and carcinogenicity (Crooke et al. 2006; Jerry et al. 2018). These studies emphasize the importance of estrogen metabolism in the occurrence and development of breast cancer.

Pituitary tumor transforming gene 1 (*PTTG1*) is known as an oncogene associated with tumorigenesis (Yoon et al. 2012; Liang et al. 2015; Wondergem et al. 2012). Previous studies have demonstrated that *PTTG1* promotes cancer cell proliferation, migration, and invasion (Li et al. 2013; Huang et al. 2014). For instance, Li and colleagues have reported that PTTG1 promotes cell proliferation, migration, and invasion in the non-small cell lung cancer cells (Li et al. 2013). Another study has identified several microRNAs and p53 as the targets in PTTG1 mediated tumorigenesis (Liang et al. 2015). In 2012, Yoon and colleagues have demonstrated that PTTG1 is associated with the epithelial-mesenchymal transition (EMT) and is also correlated with the regulation of cancer stem cells in breast cancer (Yoon et al. 2012). In 2015, another study initiated by Xiea finds that PTTG1 promotes the development of breast cancer by the regulation of *p27*, a gene that regulates the cell cycle (Huang et al. 2014). Although many studies have demonstrated the roles of PTTG1 in breast cancer, the underlying mechanisms are still unclear. To our knowledge, the connections between PTTG1 and estrogen in breast cancer are also unknown. Herein, in the present study, for the first time, we investigated whether the levels of PTTG1 were associated with estrogen.

## Materials and methods

### Cell line and cell culture

Breast cancer cell lines including MCF7 and T47D were purchased from the American Type Culture Collection (ATCC, Manassas, VA, USA) and cultured in the recommended complete medium supplied with 10% fetal bovine serum (FBS, Gibco, Grand Island, NY), 100 μg/ml streptomycin (Sangon, Shanghai, China), and 100 IU/ml penicillin (Sangon) at 37 °C in the presence of 5% CO_2_. MCF7 cells were cultured in complete Eagle’s minimum essential medium supplied with 10% FBS, 0.01 mg/ml human recombinant insulin, 100 μg/ml streptomycin, and 100 IU/ml penicillin. T47D cells were cultured with RPMI-1640 medium supplied with 10% FBS and 0.2 units/ml bovine insulin, 100 μg/ml streptomycin, and 100 IU/ml penicillin.

### Construction of stable cell lines

In the present study, PTTG1 or ESR1 knockdown cells were constructed using shRNA transfection, the PLKO.1 was used as control vector. In brief, when the cells reach 50–70% confluency, the cells were transfected with plasmids containing shRNA sequence and transfection reagent complex (Lipofectamine 3000, Thermo Fisher, Waltham, MA, USA). After that, the cells were incubated for another 24 h. Puromycin (Sangon) solution was used to select the stably transfected cells. qPCR was then applied to ensure the efficiency of PTTG1 knockdown. The sequence of shRNAs were as follows: shPTTG1: CCG GCC TTC AAT CAA AGC CTT AGA TCT CGA GAT CTA AGG CTT TGA TTG AAG GTT TTT. shESR1: CCG GAG CAC CCT GAA GTC TCT GGA ACT CGA GTT CCA GAG ACT TCA GGG TGC TTT TTT.

Additionally, PTTG1 overexpressed cells were constructed using the pEGFP vector. In brief, after the DNA fragment containing PTTG sequences were inserted into the vector. The cells were transfected with an engineered vector followed by the selection of antibiotics. qPCR was applied to ensure whether the cells overexpressed with PTTG.

### Cell viability assays

In the present study, cell count and 3-(4,5-Dimethylthiazol-2-yl)-2,5-diphenyltetrazolium bromide (MTT) assays were used to evaluate cell proliferation. For cell count assay, after the cells were treated with shRNAs, trypan blue (Sangon, Shanghai, China) solutions were used and cell numbers were calculated. For MTT assay, MTT solution (Sigma, St. Louis, MO, USA) was added into each well and incubated in 37 °C for 2 h. After that, dimethyl sulfoxide (Sigma) was added into each well to dissolve the MTT formazan. Next, the plate was then read using a microplate reader with wavelength at 570 nm and 690 nm.

### Cell cycle analysis

To evaluate the effects of PTTG1 on the cell cycle, propidium iodide (PI, Sigma) staining was performed followed by flow cytometry analysis. In brief, after the cells were harvested, 70% ethanol was used to fix the cells. Next, the cells were washed with PBS twice and then treated with RNase to remove RNA in the cells. PI solution was used to stain the cells and then flow cytometry was applied to collect the data (BD FACSVerse Flow Cytometer, San Jose, CA, USA). Flowjo software (FlowJo, Ashland, OR, USA) was applied to analyze the data.

### Estrogen deprivation

To explore the effects of estrogen deprivation on the expressions of PTTG1, the cells were deprived of estrogen according to a previously reported method (Xie et al. 2017). In brief, the cells were cultured in a medium containing exogenous estrogen for 7 days. After that, the medium was replaced by a phenol red-free medium containing 5% FBS in the absence of estrogen.

### qPCR

RNA extraction kit (Trizol, Invitrogen Pleasanton, CA, USA) was used to isolate RNA from the cells, according to the document of the manufacturers. Reverse transcriptase (Transgen, Beijing, China) was used in the RT reaction. The realtime-PCR was analysed with SYBR green (Transgen, Beijing, China) in Agilent Mx3000P (Santa Clara, CA, USA). The Melt curves were used to analyze the accuracy. The expressions of each gene were calculated using 2^-△△Ct^ values. The mRNA expression values of target genes were normalized to that of *GAPDH*.

### Luciferase assays

Luciferase assays were applied to investigate whether the promoter sites of PTTG1 exist ER binding sites. The DNA fragment containing the promoter of PTTG1 was annealed and then inserted into pGL3 basic backbone (Promega, Madison, WI, USA), which has luciferase sequence. Additionally, we further introduced mutations in the ER binding sites including mutation of two binding sites separately and mutation of two binding sites simultaneously. After that, we seeded the cells in the microplates. When the cells reach 60–70% confluency, the cells were co-transfected with engineered pGL3 plasmids and estrogen*.* The activities of luciferase were determined after the transfection of 24 h.

### Statistical analysis

Data were shown as mean ± S.D. All biochemical experiments were repeated at least 3 times. One-way analysis of variance with multiple comparisons and Student-Newman-Keuls (SNK) test were performed. A *p*-value that less than 0.05 was thought as a statistical significance between the two groups.

## Results

### The levels of PTTG1 were associated with poor survival rates in breast cancer

**Fig. 1:**
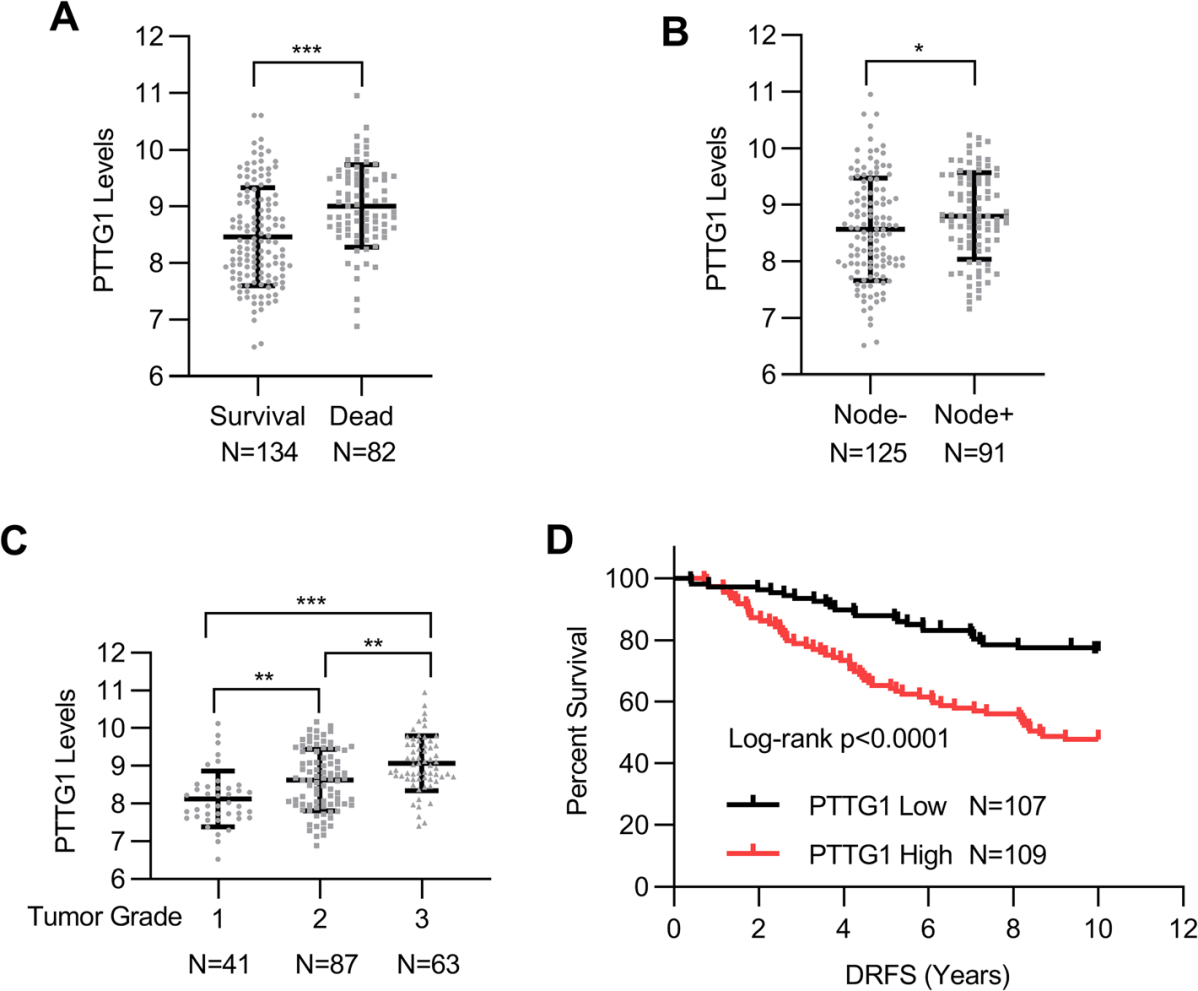
The levels of PTTG1 were associated with poor survival rates in breast cancer. To analyze the relationship between PTTG1 and survival rate of patients with breast cancer, GSE22220 from the GEO dataset was applied. The dataset includes live and deceased cohorts according to the survival status of patients. The mRNA expression of PTTG1 was compared between live and deceased cohorts. **a** The mRNA levels of PTTG1 in survival patients and deceased patients. **b** The mRNA levels of PTTG1 in patients with or without lymphatic metastasis. **c** The mRNA levels of PTTG1 in patients with different tumor grades. **d** Kaplan–Meier plots were used to analyze stratification of survival rate in cancer patients according to their PTTG1 levels. Data were represented as mean ± S.D. *P < 0.05, **P < 0.01, ***P < 0.001, ns indicates no significance

First, we explored the relationship between PTTG1 mRNA expression pattern and survival status of patients with breast cancer using the GSE22220 dataset. The dataset includes live and deceased cohorts according to the survival status of patients. The mRNA expression of PTTG1 was compared between live and deceased cohorts. Interestingly, we observed the mRNA levels of PTTG1 in survival patients were much higher than those in the deceased patients (Fig. 1a, *p* < 0.001). Besides, the patients with lymphatic metastasis showed higher PTTG1 mRNA levels than those in the patients without lymphatic metastasis (Fig. 1b, *p* < 0.05). We also found that the mRNA levels PTTG1were positively associated the tumor grade, which is supported by patients with severe tumor grade have high mRNA levels of PTTG1 (Fig. 1c). Kaplan–Meier plots further demonstrated that patients with low expression of PTTG1 possessed a higher survival rate as compared to the patients with high expression of PTTG1 (Fig. 1d).

### PTTG1 affected the cell viabilities of breast cancer cells

**Fig. 2:**
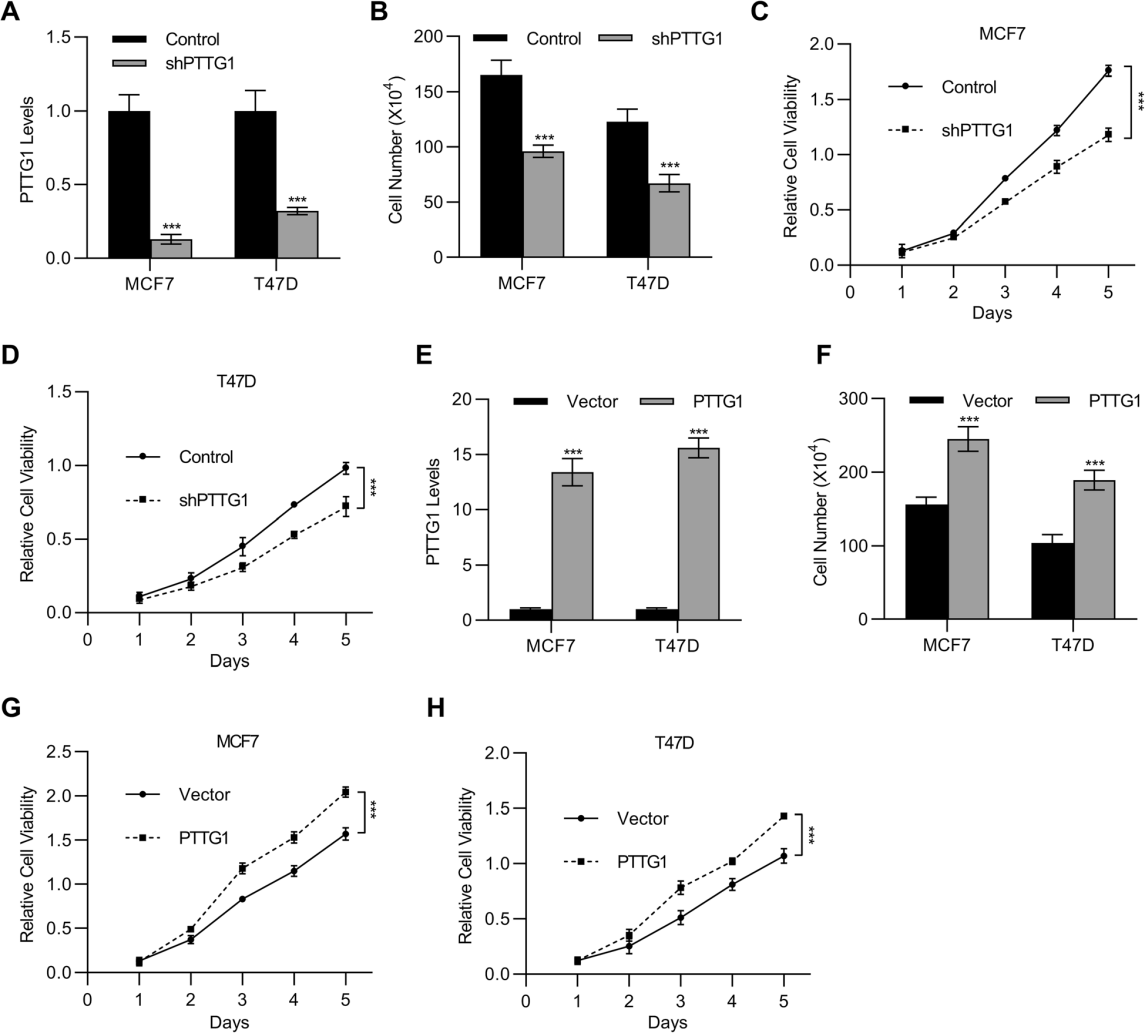
PTTG1 promoted the proliferation of breast cancer cells. **a** qPCR was employed to detect the mRNA levels of PTTG1 in the cells including MCF7 and T47D that were transfected with shPTTG1 or negative control shRNA. *n* = 3. After that, (**b-d**) Cell count (**b**) or MTT (**c-d)** assay was used to determine the cell viabilities. *n* = 7. Additionally, (**e**) cells including MCF7 and T47D were used to establish PTTG1 overexpression cell lines. qPCR was employed to detect the mRNA levels of PTTG1 in the cells. *n* = 3. **f-h** Cell count (**f**) or MTT (**g-h**) assay was used to determine the cell viabilities. *n* = 7. Data are represented as mean ± S.D. ***P < 0.001

We further analyzed the effects of PTTG1 on cell viabilities. PTTG1 shRNAs were successfully employed to knock down the expressions of PTTG1 in the breast cancer cell line including MCF7 and T47D (Fig. 2a). Next, cell viabilities were determined. The results demonstrated that the knockdown of PTTG1 resulted in a significant decrease of cell viabilities in the MCF7 and T47D cells (Fig. 2b-d, *p* < 0.001).

In addition to the construction of PTTG1 knockdown cell lines, PTTG1 overexpressed cell lines were successfully established (Fig. 2e). Interestingly, we observed the cell viabilities is negatively correlated to the PTTG1 expressions. The breast cancer cells that were transfected PTTG1 overexpression plasmids have lower cell viabilities (Fig. 2f-h) as compared to the breast cancer cells in the absence of PTTG1 plasmids transfection. Overall, the results demonstrated that PTTG1 effected the cell viabilities of breast cancer cells.

### PTTG1 expression was regulated by the levels of estrogen

**Fig. 3:**
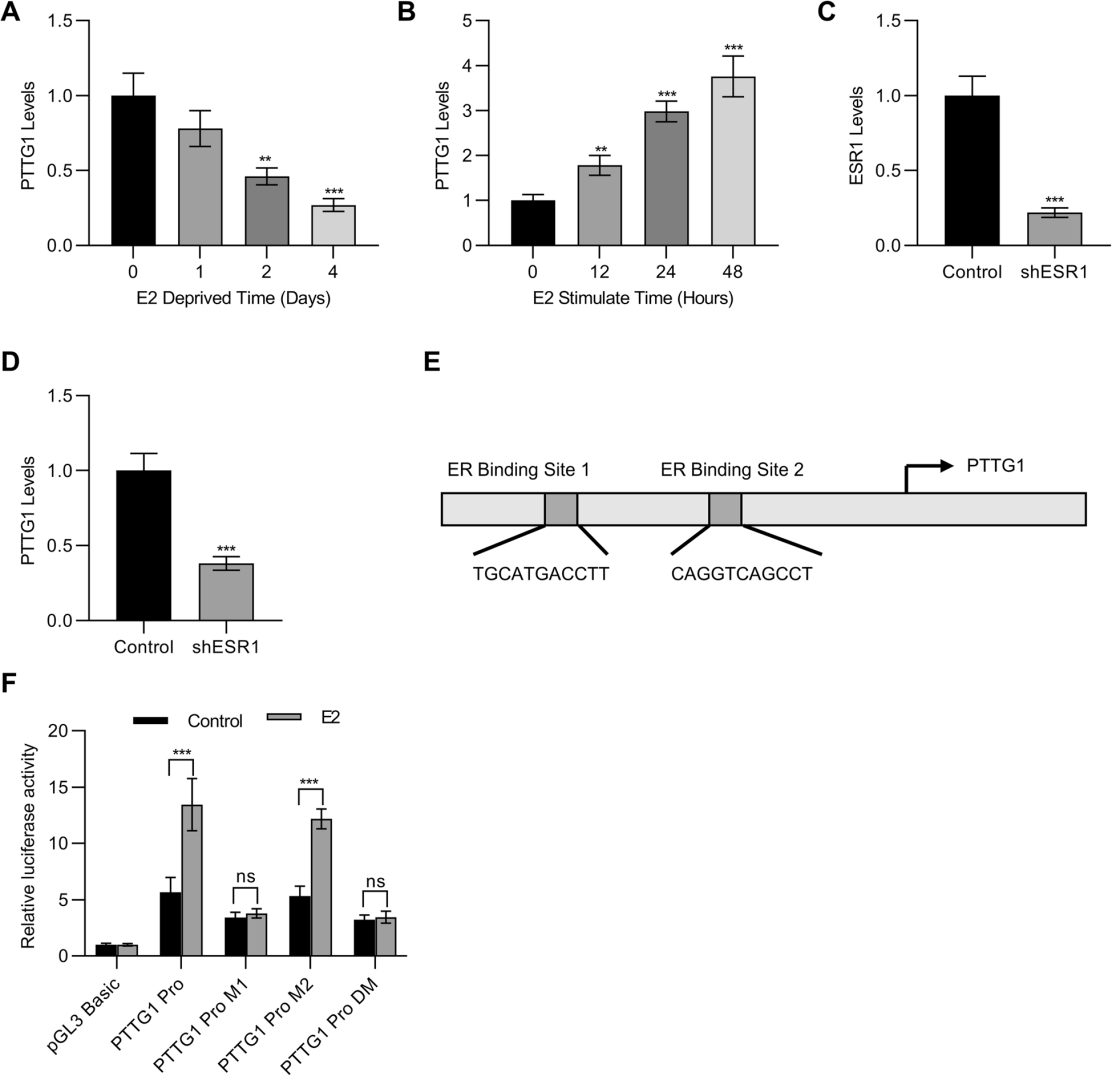
PTTG1 expression was regulated by the levels of estrogen. **a-b** qPCR was employed to detect the mRNA levels of PTTG1 in MCF7 cells in the presence or absence of estrogen (100 nM). **c-d** shRNA was used to knock down the ESR1 and qPCR was applied to examine the mRNA levels of ESR1 and PTTG1 in the cells. **e** ER binding sites were predicted using rVista (https://rvista.dcode.org/) and two possible binding sites were found. **f** Mutations were introduced in the ER binding sites and luciferase reporter assay was performed to confirm the ER binding sites on the promoter on the PTTG1.MCF7 cells were co-transfected with plasmids and vehicles or estrogen. *n* = 3 for all experiments. Data were represented as mean ± S.D. **P < 0.01, ***P < 0.001, ns indicates no significance

We next explored the effects of estrogen on the expressions of PTTG1. Interestingly, we observed the mRNA levels of *PTTG1* were decreased in the absence of estrogen (Fig. 3a), whereas the mRNA levels of *PTTG1* were increased with the extra estrogen stimulation (Fig. 3b). These results supported that the expressions of *PTTG1* were associated with estrogen. To confirm the relationship between PTTG1 and estrogen, we further applied shRNA to knockdown the estrogen receptor 1 (*ESR1*) (Fig. 3c). The results demonstrated that the knockdown of *ESR1* resulted in a significant decrease of *PTTG1* (Fig. 3d). Furthermore, we analyzed the sequence of *PTTG1* promoter using rVista (https://rvista.dcode.org/) and found two possible ER binding sites (Fig. 3e). After we introduced mutations in these ER binding sites, luciferase reporter assays were then performed. The results demonstrated that luciferase activities were significantly increased with the introduction of M2 site mutations. Therefore, it is likely that the estrogen receptor bound to the M1 site (Fig. 3f), which is an estrogen responsive site.

### The levels of PTTG1 were associated with tamoxifen resistance of breast cancer cells

**Fig. 4:**
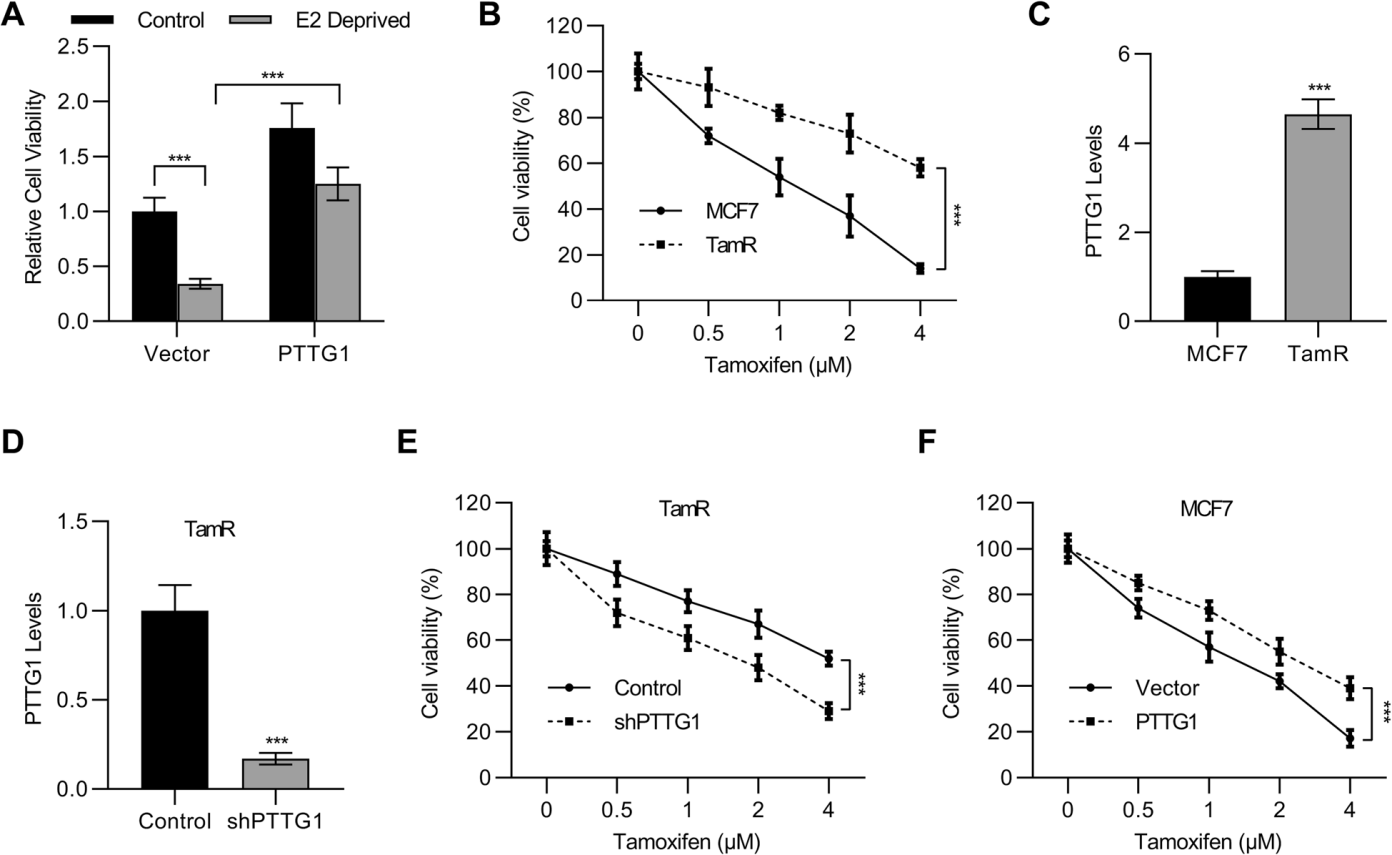
The levels of PTTG1 were associated with tamoxifen resistance of breast cancer cells. **a** MTT was used to determine cell viabilities of MCF7 cells that were transfected with vector or plasmids containing PTTG1 sequence. *n* = 6. **b** Cell viabilities of MCF7 or tamoxifen resistant (TamR) cells were also determined. *n* = 6. **c-d** qPCR was employed to determine the mRNA levels of PTTG1 in the MCF7 cells, TamR cells, and TamR cells that were transfected with shPTTG1 or control shRNA. *n* = 3. **e-f** MTT was used to determine the cell viabilities of TamR cells that were transfected with shPTTG1 or control shRNA and MCF7 cells that were transfected with vector or plasmids containing PTTG1 sequence. *n* = 6. Data were represented as mean ± S.D. ***P < 0.001

We next explored the relationship between PTTG1 and tamoxifen resistance. First, we determined the cell viabilities of MCF7 cells or PTTG1 overexpressed MCF7 cells in the presence or absence of estrogen. The results demonstrated that the cell viabilities of cells were significantly decreased in the absence of estrogen (Fig. 4a). Additionally, the cell viabilities of MCF7 cells were recovered after the transfection of PTTG1, indicating estrogen deprivation has fewer effects on cell proliferation due to PTTG1 overexpression. Second, we compared the cell viabilities between tamoxifen-resistant cells (TamR) and MCF7 cells. The results demonstrated the cell viabilities of TamR were significantly increased as compared with MCF7 cells (Fig. 4b). Interestingly, we found the levels of PTTG1 were higher in TamR cells (Fig. 4c), indicating a possible association between PTTG1 and tamoxifen resistance.

To confirm the relationship between PTTG1 and tamoxifen resistance. Next, PTTG1 was successfully knocked down in the TamR cells (Fig. 4d). We observed that the knockdown of PTTG1 resulted in a decrease of cell viabilities in the presence of tamoxifen, whereas overexpression of PTTG1 further promoted the cell viabilities in the presence of tamoxifen (Fig. 4e and f). These results demonstrated that the levels of PTTG1 were associated with tamoxifen sensitivities of breast cancer cells.

### PTTG1 regulated the expressions of CCNA2 and CCNB2 in the breast cancer cells

**Fig. 5:**
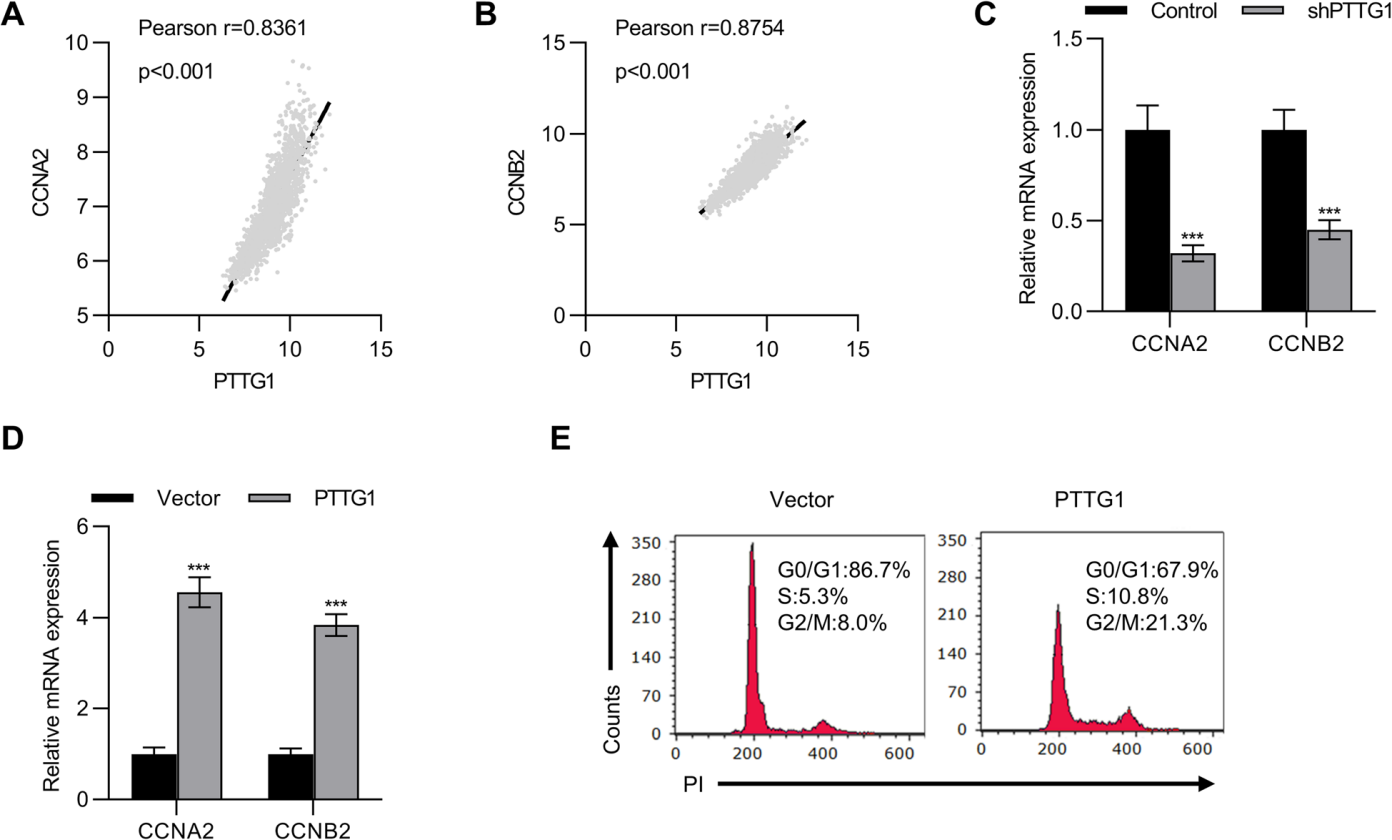
PTTG1 regulated the expressions of CCNA2 and CCNB2 in the breast cancer cells. **a-b** TCGA breast cancer dataset was used to explore the correlation between PTTG1 and genes including CCNA2 and CCNB2. **c-d** qPCR was employed to determine the mRNA levels of CCNA2 and CCNB2 in the PTTG1-knockdown and -overexpression cells. *n* = 3. **e** PI staining was performed and flow cytometry was used to analyze the effects of PPTG1 on the cell cycle. Data were represented as mean ± S.D. ***P < 0.001

Finally, we explored the possible targets of PTTG1 in breast cancer. By using the TCGA breast cancer dataset, two genes including CCNA2 and CCNB2 were identified to be associated with the PTTG1(Fig. 5a and b). We then detected the mRNA expressions of CCNA2 and CCNB2 in the PTTG1 knockdown and overexpression cells. The results demonstrated that the mRNA expressions of CCNA2 and CCNB2 were decreased in the PTTG1 knockdown cells, whereas the PTTG1 overexpression cells showed the increase of the mRNA levels of CCNA2 and CCNB2 (Fig. 5c and d). It is known that genes including PTTG1, CCNA2, and CCNB2 are associated with the cell cycle. We then explored the effects of PTTG1 overexpression on the cells. Overexpression of PTTG1 led to more cells distributed in the S phase, with an increase of 10.8%. However, there are only 5.3% of cells that were transfected with vector in the S phase (Fig. 5e).

## Discussion

Previous studies have revealed the oncogenic roles of PTTG1 in the tumorigenesis of many types of cancers including non-small cell lung cancer, hepatoma, prostate cancer, and breast cancer (Yoon et al. 2012; Liang et al. 2015; Li et al. 2013; Huang et al. 2014; Chen et al. 2006). In 2006, PTTG1 was identified as one of the mRNA biomarkers in patients with breast cancer (Chen et al. 2006). In the present study, we investigated the mRNA expression patterns of PTTG1 in patients with different survival status. Interestingly, we found the mRNA levels of PTTG1 are associated with lymphatic metastasis, tumor grade, and survival rates. The mRNA levels of PTTG1 are increased in higher tumor grade as compared to the lower tumor grade. Kaplan–Meier plots demonstrated that patients with low expression of PTTG1 possessed a higher survival rate as compared to the patients with high expression of PTTG1. These results supported that the levels of PTTG1 were associated with poor survival rates in breast cancer. Our results are in part consistent with a recent report, in which Qi and colleagues identified overexpression of PTTG1 might serve as diagnosis and poor prognosis biomarkers in breast cancer (Qi et al. 2019).

Our in vitro study further supported the oncogenic roles of PTTG1 in breast cancer. As shown in Fig. 2a and b, the knockdown of PTTG1 resulted in a significant decrease of cell viabilities in the MCF7 and T47D cells. However, the breast cancer cells with PTTG1 overexpression showed lower cell viability. These results are consistent with the previous findings shown that PTTG1 mediates cell proliferation (Yoon et al. 2012; Li et al. 2013).

The importance of estrogen has been well defined in the occurrence and development of breast cancer (Blander 2006; Crooke et al. 2006). Studies have demonstrated that high levels of estrogen are one of the major risk factors for breast cancer (Crooke et al. 2006; Horwitz and McGuire 1979). Aberrant activation of estrogen receptor signaling pathways induces the production of oxidative metabolites and the mutations of genes, therefore contributing to the DNA damage and tumorigenesis (Blander 2006; Pietras and Marquez-Garban 2007). However, the relationship between PTTG1 and estrogen is still unknown. Therefore, the present study was designed to explore the association between PTTG1 and estrogen. The results demonstrated that the mRNA levels of PTTG1 were decreased in the absence of estrogen. However, the mRNA levels of PTTG1 were increased with the stimulation by extra estrogen. These results supported the connection between PTTG1 and estrogen. To confirm the relationship between PTTG1 and estrogen, we further applied shRNA to knockdown the ESR1. The results demonstrated that the knockdown of ESR1 resulted in a significant decrease of PTTG1. Moreover, the present study also verified an estrogen response site in the PTTG1 promoter, indicating that estrogen regulated the expressions of PTTG1 through binding to the estrogen responsive site of PTTG1 promoter.

Previous study has reported that tamoxifen resistance occurs in more than half of patients with advanced breast cancer with estrogen receptor-positive (Dorssers et al. 2001). Therefore, we further explore the effects of PTTG1 on the tamoxifen resistance. TamR and normal breast cancer MCF7 cells were used. The results demonstrated that the levels of PTTG1 were associated with tamoxifen sensitivities of breast cancer cells. In particular, we found the levels of PTTG1 were higher in TamR cells. Apart from that, we also observed that the knockdown of PTTG1 resulted in a decrease of cell viabilities in the presence of tamoxifen, whereas overexpression of PTTG1 further promoted the cell viabilities in the presence of tamoxifen. These results supported that tamoxifen enhanced the effects of PTTG1 on the cell proliferation of breast cancer cells.

After the oncogenic roles of PTTG1 have been identified, many studies further elucidated the underlying mechanisms of PTTG1 in breast cancer (Yoon et al. 2012; Liang et al. 2015). For instance, Yoon and colleagues have reported that PTTG1 promotes the development of breast cancer by the regulation of EMT and cancer stem cell populations (Yoon et al. 2012). Another study has revealed that PTTG1 induces tumorigenesis of breast cancer through regulating transforming growth factor β (TGFβ) mediated signaling pathway (Zhang et al. 2015). However, there are limited studies focusing on the regulation of PTTG1 in breast cancer. In the present study, we identified that the expression patterns of PTTG1 were regulated by estrogen. Additionally, we also demonstrated that the expressions of PTTG1 were correlated to the tamoxifen resistance. These results suggested an alternative method to regulate the expressions of PTTG1.

Finally, in the present study, two targets including CCNA2 and CCNB2 were identified. CCNA2 is also known as cyclin A2 and CCNB2 is known as cyclin B2. These two genes are known as important for the regulation of the cell cycle (Casimiro et al. 2012; Nakayama and Nakayama 2006). Therefore, we speculate that the levels of PTTG1 might be associated with the cell cycle of breast cancer cells. Interestingly, the results showed that overexpression of PTTG1 led to more breast cancer cells distributed in S phase. Additionally, we also noticed that an increase of percentage of cells in G2/M from 8.0% in the vector cells to 21.3% in the PTTG1 overexpression cells. Less percentage of cells were arrested in the G1/G0 phase. Consequently, more percentage of cells were arrested in S and G2/M. These results suggested that PTTG1 affected cell viabilities in part by the regulation of cell cycle. This finding is in part consistent with a previous study, which has reported that PTTG1 affects the cell cycle in prostate cancer. However, the effects of PTTG1 on the other genes associated with the cell cycle should be explored in further studies.

## Conclusion

The present study demonstrated that the levels of PTTG1 were correlated with the survival status of patients with breast cancer. In vitro study showed that levels of PTTG1 were negatively associated with cell proliferation. Our results supported that estrogen regulated PTTG1 expression and PTTG1 reduced tamoxifen sensitivities of breast cancers. Notably, we identified that CCNA2 and CCNB2 were target genes of PTTG1 in breast cancer.

## Data Availability

Data could be obtained upon request to the corresponding author.

## Abbreviations

PTTG: pituitary tumor transforming gene
ESR: estrogen receptor
PI: propidium iodide
qPCR: quantitative polymerase chain reaction
FBS: fetal bovine serum
MTT: 3-(4,5-Dimethylthiazol-2-yl)-2,5-diphenyltetrazolium bromide

## References

[CR1] Blander CL (2006). Estrogens and breast cancer. N Engl J Med.

[CR2] Campagnoli C, Ambroggio S, Biglia N, Sismondi P (1999). Conjugated estrogens and breast cancer risk. Gynecol Endocrinol.

[CR3] Carter CL, Allen C, Henson DE (1989). Relation of tumor size, lymph node status, and survival in 24,740 breast cancer cases. Cancer.

[CR4] Casimiro MC, Crosariol M, Loro E, Li Z, Pestell RG (2012). Cyclins and cell cycle control in cancer and disease. Genes Cancer.

[CR5] Chen CC (2006). Combination of multiple mRNA markers (PTTG1, Survivin, UbcH10 and TK1) in the diagnosis of Taiwanese patients with breast cancer by membrane array. Oncology.

[CR6] Crooke PS (2006). Estrogens, enzyme variants, and breast cancer: a risk model. Cancer Epidemiol Biomark Prev.

[CR7] Dorssers LC (2001). Tamoxifen resistance in breast cancer. Drugs.

[CR8] Eismann J, et al. Interdisciplinary Management of Transgender Individuals at risk for breast cancer: case reports and review of the literature. Clin Breast Cancer. 2019;19:e12–e19. 10.1016/j.clbc.2018.11.007 PMC7083129 30527351

[CR9] Friedenreich CM (2001). Review of anthrocpometric factors and breast cancer risk. Eur J Cancer Prev.

[CR10] Giordano SH (2004). Is breast cancer survival improving? Trends in survival for patients with recurrent breast cancer diagnosed from 1974 through 2000. Cancer: Interdiscip Int J Am Cancer Soc.

[CR11] Gruber CJ, Tschugguel W, Schneeberger C, Huber JC (2002). Production and actions of estrogens. N Engl J Med.

[CR12] Gucalp A (2019). Male breast cancer: a disease distinct from female breast cancer. Breast Cancer Res Treat.

[CR13] Guo H, Gao X-H, Liu C, Li J-H (2018). An unusual localised pigmented skin lesion on the nipple-areola complex. Bmj.

[CR14] Horwitz KB, McGuire WL (1979). Estrogen control of progesterone receptor induction in human breast cancer: role of nuclear estrogen receptor. Adv Exp Med Biol.

[CR15] Huang S, Liao Q, Li L, Xin D (2014). PTTG1 inhibits SMAD3 in prostate cancer cells to promote their proliferation. Tumour Biol.

[CR16] Hulka BS, Moorman PG (2008). Reprint of breast cancer: hormones and other risk factors. Maturitas.

[CR17] Jerry DJ (2018). Genetic variation in sensitivity to estrogens and breast cancer risk. Mamm Genome.

[CR18] Li H (2013). PTTG1 promotes migration and invasion of human non-small cell lung cancer cells and is modulated by miR-186. Carcinogenesis.

[CR19] Liang HQ (2015). The PTTG1-targeting miRNAs miR-329, miR-300, miR-381, and miR-655 inhibit pituitary tumor cell tumorigenesis and are involved in a p53/PTTG1 regulation feedback loop. Oncotarget.

[CR20] Lin C-H, et al. Contrasting epidemiology and clinicopathology of female breast cancer in Asians versus the US population. JNCI. 2019;111:1298–306. 10.1093/jnci/djz090 PMC6910158 31093668

[CR21] Nakayama KI, Nakayama K (2006). Ubiquitin ligases: cell-cycle control and cancer. Nat Rev Cancer.

[CR22] Pietras RJ, Marquez-Garban DC (2007). Membrane-associated estrogen receptor signaling pathways in human cancers. Clin Cancer Res.

[CR23] Qi L (2019). Significant prognostic values of differentially expressed-aberrantly methylated hub genes in breast cancer. J Cancer.

[CR24] Siegel RL, Miller KD, Jemal A (2019). Cancer statistics, 2019. CA Cancer J Clin.

[CR25] Wondergem B (2012). Expression of the PTTG1 oncogene is associated with aggressive clear cell renal cell carcinoma. Cancer Res.

[CR26] Xie G (2017). UTX promotes hormonally responsive breast carcinogenesis through feed-forward transcription regulation with estrogen receptor. Oncogene.

[CR27] Yoon CH (2012). PTTG1 oncogene promotes tumor malignancy via epithelial to mesenchymal transition and expansion of cancer stem cell population. J Biol Chem.

[CR28] Zhang G, Zhao Q, Yu S, Lin R, Yi X (2015). RETRACTED ARTICLE: Pttg1 inhibits TGFβ signaling in breast cancer cells to promote their growth. Tumor Biol.

